# Complications during mechanical ventilation—A pediatric intensive care perspective

**DOI:** 10.3389/fmed.2023.1016316

**Published:** 2023-02-01

**Authors:** Shawn Sood, Hammad A. Ganatra, Francesca Perez Marques, Travis R. Langner

**Affiliations:** Division of Pediatric Critical Care, Kansas University Medical Center, Kansas City, KS, United States

**Keywords:** pediatric, mechanical ventilation, pediatric intensive care, complications, atelectasis, subglottic stenosis, delirium, myopathy

## Abstract

Mechanical ventilation is a common procedure performed in pediatric intensive care units, with over 20% of patients requiring invasive ventilator support. The most common indication for endotracheal intubation and ventilation in the pediatric population is respiratory failure either due to respiratory embarrassment or neurologic pathology. Despite the use of ventilation modes that are lung protective in the pediatric population, complications of mechanical ventilation occur frequently. These include atelectasis, post-extubation stridor, perioral tissue damage, ventilator associated pneumonia, mucus plugging, pneumothorax, pneumomediastinum, and ICU neuromyopathy. The purpose of this review is to discuss the risk factors, presentation and management of complications associated with mechanical ventilation in the pediatric population.

## 1. Introduction

Mechanical ventilation is used routinely in pediatric intensive care units (PICU), with over 20% of patients requiring invasive ventilator support (1). While respiratory disease is a major indication for invasive mechanical ventilation, there are also numerous non-respiratory indications for mechanical ventilation, including neurological and neuromuscular pathology, congenital heart disease, hemodynamic shock, and postoperative care and pain management. Pediatric patients are currently ventilated with a lower tidal volume and lower peak inspiratory pressure when compared to clinical practice from 30 years ago (2). Despite the widespread use of lung-protective ventilation strategies in the pediatric population, complications of mechanical ventilation occur frequently, and pediatric specialists need to be proficient in identifying, preventing, and treating these complications. Below we discuss the etiology, presentation, and management of complications during the act of mechanical ventilation in the pediatric population.

## 2. Unique anatomical and physiological features of pediatric patients

Pediatric patients are anatomically and physiologically different from adults. In the pediatric population, lung volume increases at a greater rate than the increase in airway diameter which is a precipitating factor in why infants are especially prone to hyperinflation and air trapping in circumstances of airway narrowing or elevated resistance, such as in bronchiolitis. In infants, ventilatory capacity is limited by mechanical disadvantages, including dependence on the diaphragm as the main muscular driver of respiratory effort and relatively greater chest wall compliance. As a result, the pediatric patient must expend more energy to generate the same tidal volume than an adult individual as significant effort of breathing is lost in distortion of the rib cage. The chest wall in pediatric patients has significantly lower elastic recoil thereby putting them at greater risk of atelectasis as major percent of tidal breathing occurs within the range of the lung’s closing capacity. Additionally, infants have a reduced ability to generate muscle force due to the shape of their rib cage, reduced muscle mass, increased basal metabolic rate, diminished nutritional reserve and lower oxidative capacity than healthy adults (3).

## 3. Pediatric complications of mechanical ventilation

### 3.1. Atelectasis

Atelectasis describes a state of volume loss and non-aerated region of an otherwise normal region of the lung parenchyma. Atelectasis is the most frequent complication of mechanical ventilation in pediatric patients (4). The implementation of lung protective ventilation strategies with low tidal volume ventilation may contribute to the increased development of atelectasis secondary to underinflation of alveolar units. The management of atelectasis is increasing airway pressure to a level higher than the critical opening pressure. However, in most pediatric lung diseases the critical opening pressure is elevated in a heterogeneous manner, and using positive pressure to maintain open recruitment of injured lung segments can potentially result in overdistention of healthier lung segments (5). Positive end-expiratory pressure (PEEP) is used frequently to treat atelectasis by successfully overcoming the closing pressure of smaller airways. Recruitment maneuvers through sustained inflation or stepwise increases in PEEP have demonstrated improvements in lung reaeration, however, the optimum method of recruitment remains under discussion (6).

Patients with refractory atelectasis may require bronchoscopy. However, the benefits of bronchoscopy must be carefully weighed against the risks. Many PICU patients may be too unstable to withstand the decreases in PaO2 and elevation in PaCO2 that often are associated with bronchoscopic intervention. Despite the utilization of adequate sedation, bronchoscopy leads to an elevation in intracranial pressure and should be weighed accordingly in patients with traumatic brain injury or other intracranial pathologies. While bronchoscopy may be beneficial to alleviating mucus plugging, it does not have significant utility in managing atelectasis from other causes such as pleural effusion and diaphragmatic dysfunction.

Mechanical chest physiotherapy including chest wall percussion or vibration is also frequently utilized as a treatment modality for pulmonary atelectasis in children (7). However, strong data for its continued use is lacking. As an alternative, intermittent percussive ventilation (IPV) has been demonstrated to be superior to chest physiotherapy in a controlled pediatric trial (8).

More recently, lung ultrasound has been proposed as an additional tool for identification of pulmonary atelectasis in children, as well as monitoring for improvement of atelectasis during recruitment maneuvers (9, 10). However, most of the published data is based on small samples of specialized pediatric populations, and randomized trials on wide section of pediatric ICU patients is needed before ultrasound can replace radiographic modalities for diagnosing and monitoring atelectasis.

### 3.2. Perioral tissue damage

The most common cause of medical device-related pressure ulcer are the mucosal pressure ulcers caused by prolonged pressure from endotracheal tubes (11). When compared to adult ICUs, the duration of intubation and invasive mechanical ventilation is longer in pediatric ICUs and the threshold for tracheostomy is much higher. This is understandable given the greater regenerative and healing capacity of pediatric lung tissues, but unfortunately predisposes to higher incidence of oral pressure injuries. Frequent monitoring and proper repositioning of mechanical pressure on the oral mucosa is an effective preventive strategy for reducing the development and advancement of mucocutaneous complications. Pressure-induced injury to the oropharyngeal tissues secondary to laryngeal mask airway insertion, forceful suctioning of posterior teeth, spasm of the masseter muscle secondary to hypothermia induced shivering and biting forces against antagonist teeth are additional etiologies of perioral tissue damage (12). Reagrding dental traumas, if the pediatric patient has a loose tooth, suturing the affected tooth to the adjacent sound tooth may prevent avulsion and subsequent aspiration (12, 13). In our personal clinical practice, we have found physical and occupational therapists to be an exceptional resource for the team as they assist and educate bedside nurses with adequate alternating positioning of intubated children to prevent intraoral pressure injuries.

A potential preventive strategy in the future could be the use of SecureTube™ that is under trial and development by Deshpande et al. (14). This re-imagined endotracheal tube has been designed primarily to prevent unplanned extubations among children, and incorporates a swivel that allows the endotracheal tube to move in the direction of patient head movement, instead of against it. This security feature invariably results in reduced frictional contact between the SecureTube™ and oral tissue and it would be interesting to see if future trials evaluate for and demonstrate potentially lower oral pressure injuries.

### 3.3. Ventilator associated pneumonia

After blood stream infections, ventilator associated pneumonia is the second most frequently occurring nosocomial infection in the PICU (15). The origin of ventilator associated pneumonia is likely the result of micro-aspirations. The most accepted definition of ventilator associated pneumonia is pneumonia occurring after the patient has been intubated and received mechanical ventilation for at least 48 hours. The initial diagnosis is based on clinical suspicion and the presence of at least one of the following on two or more serial chest radiographs: new or progressive radiographic infiltrates, consolidation, cavitation, and pneumatoceles in an infant 1-year-old or above. Additionally, the standard diagnostic criteria include at least two of the following (or three in patients under the age of 12 years): hyperthermia or hypothermia; change in character or volume of sputum production, or increased requirement for secretion clearance; new onset cough or worsening of pre-existing cough, respiratory distress, tachypnea or apnea; rhonchi, wheezing, rales, or bronchial breath sounds (5, 15). Efforts to minimize ventilator associated pneumonia in the pediatric population have primarily targeted preventive strategies such as changing ventilator circuits only as needed, mouth care, medical staff hand hygiene, elevating the head of the bed, and frequent drainage of condensate in the ventilator away from the endotracheal tube, preventing aspiration by monitoring and minimizing gastric residuals, and early extubations to minimize time spent on the ventilator. Ventilator associated pneumonia mandates early antimicrobial treatment, typically starting with broad spectrum antibiotics with nosocomial organism coverage, subsequently narrowing to specific antibiotic therapy once bacterium is identified on respiratory culture.

Areas for future development would potentially include identifying biomarkers from serum and/or respiratory aspirate that can accurately and promptly identify ventilator associated pathogens. Presently, identification is based mainly on tracheal aspirate cultures and gram stains that can take up to 48 hours for speciation. Furthermore, the identification can be tricky in pediatric patients with prolonged mechanical ventilation course who may have concomitant colonization of their airways with multiple non-pathogenic organisms in addition to the pathogenic organism responsible for the episode of ventilator associated pneumonia.

### 3.4. Mucus plugging

Pediatric intensive care units patients with increased length of stays can develop critical illness myopathy and neuropathy which contributes to weakened cough and pooling of secretions. The utilization of sedatives and neuromuscular blockade exacerbates this problem by impairing muco-ciliary clearance and natural cough mechanisms. Smaller airways in the pediatric population are especially susceptible to obstruction by secretions, thereby leading to ventilation-perfusion mismatch and further worsening of respiratory failure. Acute decompensation can occur if the endotracheal tube becomes obstructed with mucus, whereby suctioning or exchange of the endotracheal tube may be necessary. Several strategies are employed to minimize and treat mucus plugging, include ventilator circuit humidification, utilization of mucolytic agents, and use of mechanical devices or techniques to mechanically disrupt mucus. Airway clearance can be accomplished through nebulization with hypertonic saline to thin out tenacious secretions that are otherwise difficult to suction. Several mechanical airway clearance techniques are commonly used in the PICU: chest physiotherapy, intermittent percussive ventilation, and cough assist. Chest physiotherapy techniques include manual percussion with hand cupping, vibration of the chest wall to loosen bronchial secretions, and postural drainage using gravity to move secretions from peripheral airways to the larger bronchi. Despite its widespread use, evidence for chest physiotherapy remains weak (7, 16). During intermittent percussive ventilation, expiratory flow exceeds inspiratory flow to propel secretions forward and can be an effective tool in pediatric patients on invasive mechanical ventilatory support with atelectasis due to mucus plugging (5, 8). Cough-assist functions by inflating the lungs with positive pressure, followed by the rapid application of negative pressure that stimulates a cough. This also generates shear forces within the airway, thereby mobilizing secretions. Based on existing practices and evidence in pediatric population, cough assist is used primarily to promote pulmonary hygiene in children with neuromuscular diseases. However, care should be taken prior to its use in critically ill children and skilled staff should be involved as it can result in lung de-recruitment if used inappropriately. Table 1 summarizes the various airway clearance modalities discussed above.

**TABLE 1:** Summary of airway clearance modalities, with mechanisms of action, and contraindications.

Modality	Mechanism	Contraindications
Intermittent percussive ventilation (IPV)	Delivers small tidal volumes at a high frequency, creating percussion forces that mobilize pulmonary secretions	• Air leak syndromes, especially in absence of chest tube • Hemoptysis • Active airborne spread infections (e.g., tuberculosis, measles)
Cough assist device (Insufflator-exsufflator)	Delivers positive pressure, quickly followed by negative pressure, thereby simulating cough mechanism and pulmonary clearance	• Untreated air leak syndromes • Hemoptysis • Recent cardiothoracic surgery • Elevated intracranial pressure • Rib fractures
Vest therapy	Mobilize secretions from bronchial walls through externally applied chest wall vibrations	•Untreated air leak syndromes • Hemoptysis • Recent chest wall incisions (surgical, traumatic, etc.) • Rib fractures • Presence of temporary pacemaker with risk of lead dislodgement • Unstable head & neck injuries • Pulmonary embolism
Chest physiotherapy (postural drainage and external clapping)	Using gravity and patient repositioning in combination with external vibration to dislodge and mobilize pulmonary secretions	• Unstable head and neck injuries • Spine injuries that preclude frequent repositioning • Rib fractures • Surgical or traumatic chest wall injuries

### 3.5. Air leak syndromes

**FIGURE 1:**
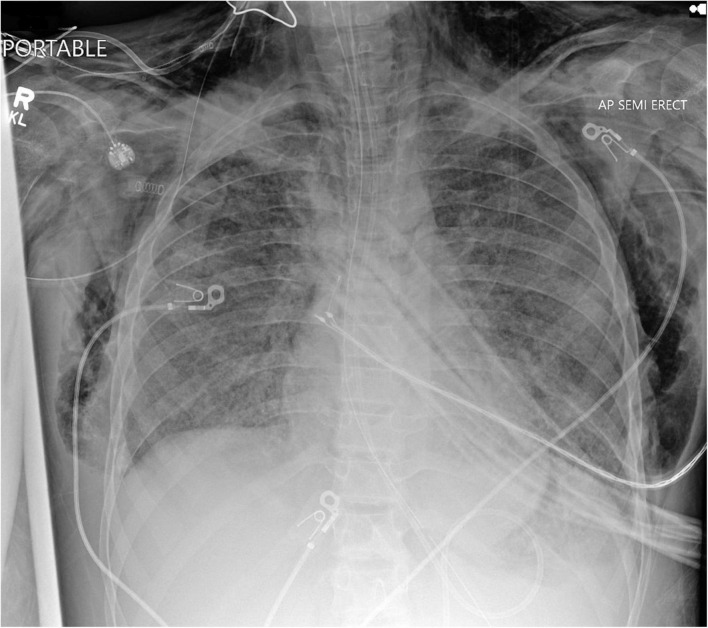
Chest radiograph of a mechanically ventilated pediatric patient, demonstrating subcutaneous emphysema und pneumomediastinum, in the absence of hemodynamic compromise.

Aggressive mechanical ventilation and resulting barotrauma or volutrauma is the most frequent etiology of air leak syndromes such as pneumothorax, pneumomediastinum, and subcutaneous emphysema in the PICU. Mechanical ventilation of children with obstructive lung physiology can also result in hyperinflation secondary to inadequate expiratory flow and thereby lead to life threatening air leak. However, with the adoption of lung protective ventilation strategies in the pediatric population, the incidence of air leak syndromes in mechanically ventilated patients has decreased. Subcutaneous emphysema is rarely clinically significant, but can be cosmetically disconcerting for parents of pediatric patients. Management of subcutaneous emphysema is mainly conservative through limiting airway pressures and allowing time for resolution of air leak. Active treatment is rarely warranted or undertaken, but there are published reports on the use of “micro-drainage” through fenestrated subcutaneous catheters in the adult population (17–19). However, no such reports are available in the pediatric population. Figure 1 shows an anonymized patient with massive subcutaneous emphysema and pneumomediastinum without any hemodynamic instability.

A large pneumothorax can lead to respiratory and hemodynamic embarrassment and treatment may require urgent needle thoracostomy and chest tube placement. In emergent situations such as acute cardiopulmonary arrest, pneumothorax is primarily a clinical diagnosis. However, in non-emergent situations where pneumothorax is suspected, chest radiographs have historically been used to confirm diagnosis. More recently, the use of point-of-care ultrasound (POCUS) has greatly enhanced the intensivist’s ability to promptly detect pneumothorax at the bedside. More extensive discussion of POCUS techniques would be beyond the scope of this review, and readers are referred to a recent review by Bhoil et al. (20). Pediatric specific literature supporting the use of POCUS in the pediatric ICU setting is steadily accumulating (21–24), but concerns persist about its heterogeneous adoption between different pediatric and neonatal centers (22).

### 3.6. ICU neuromyopathy

Mechanical ventilation for greater than 72 h leads to proteolysis triggered by oxidative stress, potentially contributing to atrophy and weakness of the diaphragm, leading to difficulty in weaning patients from mechanical ventilation (25). At rest state, respiratory muscles partake in 1–2% of basal total oxygen consumption of the body. Subsequently, as respiratory effort increases, oxygen utilization of respiratory muscles increases significantly. In a study of patients being weaned from mechanical ventilation, 24% of the total body oxygen delivery was utilized by muscles of the respiratory system, and in some cases it was more than 50% (26). This data shows that mechanically ventilated children with increased work of breathing and patient–ventilator can be afflicted by extubation failure. Ventilation with neurally adjusted ventilatory assist (NAVA) improves patient–ventilator synchrony which results in easing of the diaphragmatic workload and lower metabolic demand for the patient thereby reducing oxygen delivery demands. NAVA is a mechanical ventilation modality that determines the inspiratory airway pressure that needs to be delivered by taking into consideration the electrical depolarization of the diaphragm and is gaining more use in pediatric ICU settings. Acquired ICU neuromyopathy can prolong hospitalization. Ambulation, early physical therapy, and mobility while still receiving mechanical ventilation is important to implement in the PICU to minimize complication of critical illness myopathy and neuropathy (27).

### 3.7. Post-extubation stridor

Post-extubation stridor is deemed to be present if stridor occurs within two hours of extubation. The incidence of post-extubation stridor is approximately 5% in patients who are intubated for more than 24 hours (28). Patients with post-extubation stridor are more likely to undergo reintubation, which is associated with increased morbidity and mortality (29). Prolonged invasive mechanical ventilation may lead to laryngeal damage as pressure from the endotracheal tube is thought to cause ischemia and consequently erosion of the laryngeal mucosa. Young age, increased duration of endotracheal intubation and the use of cuffed tubes are associated with an increased risk of post-extubation stridor (30, 31). Studies have not shown an association between the occurrence of stridor and the absence of an air leak prior to extubation, however, frequent endotracheal suctioning prior to extubation has shown to be a predictor for post-extubation stridor (32). In adults, corticosteroids begun 12–24 h prior to extubation appears beneficial for patients with a high likelihood of post extubation stridor. However, using corticosteroids to prevent or treat stridor after extubation has not proven effective for the pediatric population (33). Nebulized L-epinephrine is another treatment option for post-extubation stridor in pediatric patients that has not proven to be effective but is nonetheless commonly employed. Side effects of nebulized L-epinephrine are tachycardia, pallor and an increase in blood pressure (34). If re-intubation is required, use of a smaller endotracheal tube is recommended to avoid additional trauma to the airway.

Early detection of airway edema and impending airway obstruction even before the clinical manifestation of post-extubation stridor could be crucial in improving outcomes in recently extubated children. Kikutani et al. (35) have developed a novel system that continuously monitors respiratory sounds and produces a visual representation that allows clinicians to detect sub-clinical post-extubation stridor. Although this tool needs to be validated through widespread testing, it could be invaluable in pediatric ICU setting if it allows earlier intervention with L-epinephrine nebulization and/or steroids that may have a greater impact if administered quickly, and prevent further airway instrumentation and intubation.

### 3.8. Acquired subglottic stenosis

The subglottis is the narrowest section of the airway in infants. Acquired subglottic stenosis is the most common acquired laryngeal anomaly, with the vast majority of cases attributable to prior intubation. The current risk of developing subglottic stenosis among infants mechanically ventilated for a prolonged period of time is 1% (36). Patients with long mechanical ventilation days, premature patients, and patients with gastroesophageal reflux disease are more susceptible to acquired subglottic stenosis (37). Mild forms of subglottic stenosis result in stridor & dyspnea with exertion. More severe subglottic stenosis can lead to inability to be mask ventilated. An airway may be considered critical if there is severe narrowing of the airway making mask ventilation difficult or not possible at all (38). Management for patients with mild subglottic stenosis ranges from observation to balloon dilation and endoscopic cricoid split techniques. Severe subglottic stenosis often necessitates a tracheostomy to establish a patent airway (36).

Preliminary data from *in vitro* setting has demonstrated the utility of drug-eluting endotracheal tubes that can decrease bacterial burden in the subglottic area, and theoretically decrease bacterium induced inflammation (39). Alleviating this inflammation can potentially limit scar tissue formation and thereby decrease associated subglottic stenosis, but further research is needed with *in vivo* testing to explore the full benefit of such devices. The biomedical industry should also be encouraged to engage in materials research to design endotracheal tubes with innovative and minimally noxious materials that could potentially limit subglottic scarring.

### 3.9. Vocal cord paralysis

Vocal cord dysfunction due to recurrent laryngeal nerve neuropraxia is secondary to compression of the nerve between the endotracheal tube and thyroid cartilage. Vocal cord paralysis seen after intubation is generally unilateral, with most cases comprising the left vocal cord. Mechanically ventilated patients who are less than 32 weeks in gestational age and patients who required more than five intubation attempts have a higher incidence of vocal fold paralysis. Patients present with dysphonia including hoarse voice or low-pitched cry. Laryngoscopic assessment is the gold standard for assessment of dysphonia in children. Recurrent laryngeal nerve reinnervation is a treatment option for pediatric patients with persistent unilateral vocal fold immobility (40).

### 3.10. Pediatric ICU delirium

Delirium occurs frequently in the pediatric ICU and is independently associated with mortality. Risk factors for pediatric delirium include mechanical ventilation, young age, high severity of illness and vasopressor use (41). Critically ill children who are mechanically ventilated require sedatives and analgesia. Adequate sedation facilitates ventilator synchrony and ensures comfort (42), but can be associated with ICU delirium. Implementation of universal delirium screening, early mobilization, minimization of benzodiazepine use, and utilization of a sedation weaning protocol have been shown to decrease pediatric delirium rates (43). With increasing recognition of the association of delirium with benzodiazepine use, dexmedetomidine has become an increasingly utilized sedative. Use of dexmedetomidine is limited by dose-related bradycardia. In situations where delirium does not respond to environmental changes and limitation of delirium-inducing medications, antipsychotics have been utilized (44). Concerns for the risk of QTc prolongation and extrapyramidal symptoms have limited use of antipsychotics among pediatric ICU providers.

## 4. Conclusion

Despite the adoption of lung protective strategies in the mechanical ventilation of pediatric patients, a multitude of complications still occur. Knowledge of the differing physiology of pediatric patients compared to adults, as well as the varying pathologies in PICU patients is essential to understand and minimize the risk of pulmonary complications and thereby improve outcomes in mechanically ventilated pediatric patients.

## Author contributions

SS and HG conceived and designed the project. SS wrote the first draft of the manuscript. HG revised the initial draft of the manuscript. FP and TL reviewed and revised subsequent drafts for important intellectual content. All authors approved the final version of the manuscript.
